# {4-Bromo-2-[3-(diethyl­ammonio)propyl­imino­meth­yl]phenolato}diiodidozinc(II) methanol solvate

**DOI:** 10.1107/S1600536809038446

**Published:** 2009-10-03

**Authors:** Xue-Wen Zhu, Zhi-Gang Yin, Xu-Zhao Yang, Gang-Sen Li, Chun-Xia Zhang

**Affiliations:** a Key Laboratory of Surface and Interface Science of Henan, School of Material & Chemical Engineering, Zhengzhou University of Light Industry, Zhengzhou 450002, People’s Republic of China

## Abstract

In the title complex, [ZnI_2_(C_14_H_21_BrN_2_O)]·CH_3_OH, the asymmetric unit consists of a mononuclear zinc(II) complex mol­ecule and a methanol solvent mol­ecule. The compound was derived from the zwitterionic form of the Schiff base 4-bromo-2-[3-(diethyl­amino)propyl­imino­meth­yl]phenol. The Zn^II^ atom is four-coordinated by the imine N and phenolate O atoms of the Schiff base ligand and by two iodide ions in a distorted tetra­hedral coordination. In the crystal structure, the methanol mol­ecules are linked to the Schiff base mol­ecules through N—H⋯O and O—H⋯O hydrogen bonds. One I atom is disordered over two positions in a 0.702 (19):0.298 (19) ratio.

## Related literature

For background to the chemistry of Schiff base complexes, see: Ali *et al.* (2008 ▶); Biswas *et al.* (2008 ▶); Chen *et al.* (2008 ▶); Darensbourg & Frantz (2007 ▶); Habibi *et al.* (2007 ▶); Kawamoto *et al.* (2008 ▶); Lipscomb & Sträter (1996 ▶); Tomat *et al.* (2007 ▶); Wu *et al.* (2008 ▶); Yuan *et al.* (2007 ▶). For related structures, see: Zhu (2008 ▶); Zhu & Yang (2008*a* ▶,*b* ▶,*c* ▶); Qiu (2006*a* ▶,*b* ▶); Wei *et al.* (2007 ▶); Zhu *et al.* (2007 ▶). For hydrogen-bond motifs, see: Bernstein *et al.* (1995 ▶).
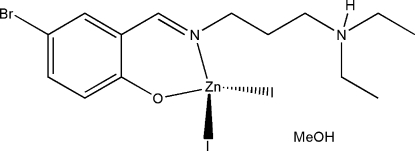


## Experimental

### 

#### Crystal data

[ZnI_2_(C_14_H_21_BrN_2_O)]·CH_4_O
*M* *_r_* = 664.45
Monoclinic, 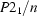

*a* = 10.869 (2) Å
*b* = 17.562 (3) Å
*c* = 11.377 (2) Å
β = 94.358 (3)°
*V* = 2165.4 (7) Å^3^
*Z* = 4
Mo *K*α radiation
μ = 5.84 mm^−1^
*T* = 298 K
0.20 × 0.20 × 0.17 mm

#### Data collection

Bruker APEXII CCD area-detector diffractometer
Absorption correction: multi-scan (*SADABS*; Sheldrick, 2004 ▶) *T*_min_ = 0.388, *T*_max_ = 0.437
14106 measured reflections
4664 independent reflections
3499 reflections with *I* > 2σ(*I*)
*R*_int_ = 0.041

#### Refinement

*R*[*F* ^2^ > 2σ(*F* ^2^)] = 0.048
*wR*(*F* ^2^) = 0.105
*S* = 1.06
4664 reflections
225 parameters
8 restraints
H atoms treated by a mixture of independent and constrained refinement
Δρ_max_ = 0.75 e Å^−3^
Δρ_min_ = −0.85 e Å^−3^

### 

Data collection: *APEX2* (Bruker, 2004 ▶); cell refinement: *SAINT* (Bruker, 2004 ▶); data reduction: *SAINT*; program(s) used to solve structure: *SHELXS97* (Sheldrick, 2008 ▶); program(s) used to refine structure: *SHELXL97* (Sheldrick, 2008 ▶); molecular graphics: *SHELXTL* (Sheldrick, 2008 ▶); software used to prepare material for publication: *SHELXTL*.

## Supplementary Material

Crystal structure: contains datablocks global, I. DOI: 10.1107/S1600536809038446/bx2241sup1.cif

Structure factors: contains datablocks I. DOI: 10.1107/S1600536809038446/bx2241Isup2.hkl

Additional supplementary materials:  crystallographic information; 3D view; checkCIF report

## Figures and Tables

**Table 1:** Selected geometric parameters (Å, °)

Zn1—O1	1.958 (4)
Zn1—N1	2.032 (5)
Zn1—I2′	2.545 (6)
Zn1—I1	2.5627 (9)
Zn1—I2	2.5768 (18)

**Table 2:** Hydrogen-bond geometry (Å, °)

*D*—H⋯*A*	*D*—H	H⋯*A*	*D*⋯*A*	*D*—H⋯*A*
O2—H2⋯O1^i^	0.82	1.86	2.640 (6)	158
N2—H2*A*⋯O2	0.91 (6)	1.81 (6)	2.716 (7)	173 (8)

Symmetry code: (i) 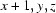.

**Fig. 1.**
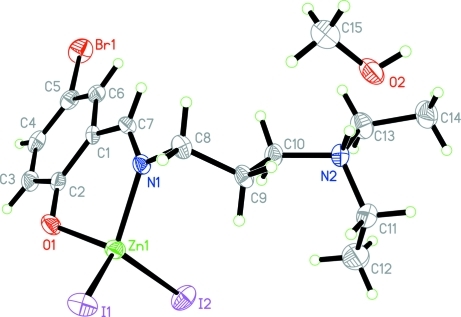
The molecular structure of the title complex, with ellipsoids drawn at the 30% probability level.

**Fig. 2.**
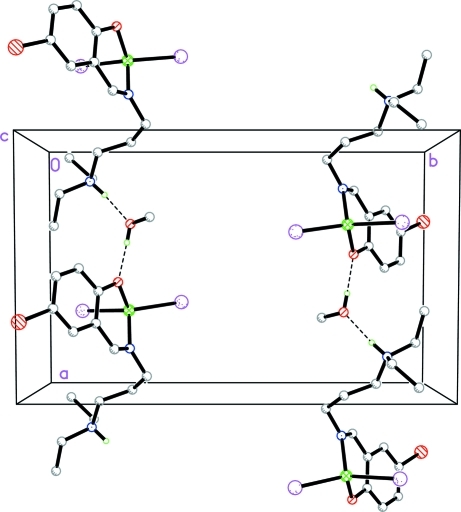
The crystal packing of the title complex.

## supplementary crystallographic information

### Comment

Schiff bases have widely been used as versatile ligands in coordination chemistry (Biswas *et al.*, 2008; Wu *et al.*, 2008; Kawamoto *et al.*, 2008; Ali *et al.*, 2008; Habibi *et al.*, 2007), and their metal complexes are of great interest in many fields (Chen *et al.*, 2008; Yuan *et al.*, 2007; Tomat *et al.*, 2007; Darensbourg & Frantz, 2007). Zinc(II) is an important element in biological systems and functions as the active site of hydrolytic enzymes, such as carboxypeptidase and carbonic anhydrase where it is in a hard-donor coordination environment of nitrogen and oxygen ligands (Lipscomb & Sträter, 1996). Recently, we have reported a few Schiff base zinc complexes (Zhu, 2008; Zhu & Yang, 2008a,b,c). In this paper, the title new zinc(II) complex, Fig. 1, is reported.

The complex consists of a mononuclear zinc(II) complex molecule and a methanol molecule. The Zn^II^ atom is four-coordinated by the imine N and phenolate O atoms of the zwitterionic form of the Schiff base ligand, and by two I^-^ ions, in a distorted tetrahedral coordination. The coordinate bond lengths (Table 1) are typical and comparable to the corresponding values observed in the Schiff base zinc complexes we reported previously and other similar Schiff base zinc complexes (Zhu *et al.*, 2007; Wei *et al.*, 2007; Qiu, 2006*a*,b). I2 atom is disordered over two positions [0.702(19/0.298 (19)].

In the crystal structure, the methanol molecules are linked to the Schiff base molecules through O—H···O and N—H···O hydrogen bonds generating a graph-set motif C^2^_2_(10) chain along [100] direction (Table 2, Fig. 2). (Bernstein *et al.*, 1995)

### Experimental

The Schiff base compound was prepared by the condensation of equimolar amounts of 5-bromosalicylaldehyde with *N*,*N*-diethylpropane-1,3-diamine in a methanol solution. The complex was prepared by the following method. To an anhydrous methanol solution (5 ml) of ZnI_2_ (31.9 mg, 0.1 mmol) was added a methanol solution (10 ml) of the Schiff base compound (31.3 mg, 0.1 mmol) with stirring. The mixture was stirred for 30 min at room temperature and filtered. Upon keeping the filtrate in air for a few days, colorless block-shaped crystals were formed.

### Refinement

H2A was located from a difference Fourier map and refined isotropically, with N—H distance restrained to 0.90 (1) Å. Other H atoms were placed in geometrically idealized positions and constrained to ride on their parent atoms, with C—H distances in the range 0.93–0.97 Å, O—H distance of 0.82 Å, and with *U*_iso_(H) = 1.2*U*_eq_(C) and 1.5*U*_eq_(methyl C and O). The I2 atom is disordered over two distinct sites with occupancies of 0.702 (2) and 0.298 (2), respectively.

### Crystal data

**Table:**

[ZnI_2_(C_14_H_21_BrN_2_O)]·CH_4_O	*F*(000) = 1264
*M**_r_* = 664.45	*D*_x_ = 2.038 Mg m^−^^3^
Monoclinic, *P*2_1_/*n*	Mo *K*α radiation, λ = 0.71073 Å
Hall symbol: -P 2yn	Cell parameters from 3253 reflections
*a* = 10.869 (2) Å	θ = 2.2–24.5°
*b* = 17.562 (3) Å	µ = 5.84 mm^−^^1^
*c* = 11.377 (2) Å	*T* = 298 K
β = 94.358 (3)°	Block, colorless
*V* = 2165.4 (7) Å^3^	0.20 × 0.20 × 0.17 mm
*Z* = 4	

### Data collection

**Table:**

Bruker APEXII CCD area-detector diffractometer	4664 independent reflections
Radiation source: fine-focus sealed tube	3499 reflections with *I* > 2σ(*I*)
graphite	*R*_int_ = 0.041
ω scans	θ_max_ = 27.0°, θ_min_ = 2.1°
Absorption correction: multi-scan (*SADABS*; Sheldrick, 2004)	*h* = −13→13
*T*_min_ = 0.388, *T*_max_ = 0.437	*k* = −22→22
14106 measured reflections	*l* = −14→13

### Refinement

**Table:**

Refinement on *F*^2^	Primary atom site location: structure-invariant direct methods
Least-squares matrix: full	Secondary atom site location: difference Fourier map
*R*[*F*^2^ > 2σ(*F*^2^)] = 0.048	Hydrogen site location: inferred from neighbouring sites
*wR*(*F*^2^) = 0.105	H atoms treated by a mixture of independent and constrained refinement
*S* = 1.06	*w* = 1/[σ^2^(*F*_o_^2^) + (0.0298*P*)^2^ + 7.4538*P*] where *P* = (*F*_o_^2^ + 2*F*_c_^2^)/3
4664 reflections	(Δ/σ)_max_ = 0.001
225 parameters	Δρ_max_ = 0.75 e Å^−^^3^
8 restraints	Δρ_min_ = −0.85 e Å^−^^3^

### Special details

**Table:**

Geometry. All esds (except the esd in the dihedral angle between two l.s. planes) are estimated using the full covariance matrix. The cell esds are taken into account individually in the estimation of esds in distances, angles and torsion angles; correlations between esds in cell parameters are only used when they are defined by crystal symmetry. An approximate (isotropic) treatment of cell esds is used for estimating esds involving l.s. planes.
Refinement. Refinement of F^2^ against ALL reflections. The weighted R-factor wR and goodness of fit S are based on F^2^, conventional R-factors R are based on F, with F set to zero for negative F^2^. The threshold expression of F^2^ > 2sigma(F^2^) is used only for calculating R-factors(gt) etc. and is not relevant to the choice of reflections for refinement. R-factors based on F^2^ are statistically about twice as large as those based on F, and R- factors based on ALL data will be even larger.

### Fractional atomic coordinates and isotropic or equivalent isotropic displacement parameters (Å^2^)

**Table:**

	*x*	*y*	*z*	*U*_iso_*/*U*_eq_	Occ. (<1)
Zn1	0.17105 (6)	0.26943 (4)	−0.06556 (7)	0.0380 (2)	
I1	0.13989 (4)	0.14098 (3)	−0.17051 (4)	0.05239 (16)	
I2	0.1766 (2)	0.39343 (11)	−0.1861 (2)	0.0541 (8)	0.702 (19)
I2'	0.1771 (8)	0.3793 (9)	−0.2109 (16)	0.118 (2)	0.298 (19)
Br1	0.19879 (8)	0.49547 (5)	0.46762 (7)	0.0630 (2)	
O1	0.0560 (4)	0.2904 (3)	0.0548 (4)	0.0443 (11)	
O2	0.8239 (4)	0.2628 (3)	−0.0180 (5)	0.0546 (13)	
H2	0.8982	0.2714	−0.0142	0.082*	
N1	0.3188 (4)	0.2626 (3)	0.0547 (4)	0.0311 (11)	
N2	0.6505 (5)	0.3665 (3)	−0.0945 (5)	0.0383 (12)	
C1	0.2134 (5)	0.3368 (3)	0.1971 (5)	0.0301 (13)	
C2	0.0899 (5)	0.3330 (4)	0.1466 (5)	0.0338 (14)	
C3	0.0011 (6)	0.3764 (4)	0.2014 (6)	0.0414 (16)	
H3	−0.0810	0.3733	0.1723	0.050*	
C4	0.0311 (6)	0.4231 (4)	0.2955 (6)	0.0431 (16)	
H4	−0.0297	0.4516	0.3284	0.052*	
C5	0.1523 (6)	0.4276 (4)	0.3417 (6)	0.0400 (15)	
C6	0.2417 (6)	0.3841 (3)	0.2958 (5)	0.0355 (14)	
H6	0.3220	0.3857	0.3302	0.043*	
C7	0.3161 (5)	0.2950 (3)	0.1550 (5)	0.0340 (14)	
H7	0.3870	0.2914	0.2059	0.041*	
C8	0.4322 (5)	0.2223 (3)	0.0264 (6)	0.0369 (14)	
H8A	0.4115	0.1707	0.0018	0.044*	
H8B	0.4888	0.2195	0.0965	0.044*	
C9	0.4942 (5)	0.2625 (3)	−0.0704 (6)	0.0349 (14)	
H9A	0.4367	0.2659	−0.1397	0.042*	
H9B	0.5644	0.2326	−0.0911	0.042*	
C10	0.5383 (5)	0.3429 (4)	−0.0348 (6)	0.0395 (15)	
H10A	0.4722	0.3789	−0.0547	0.047*	
H10B	0.5568	0.3446	0.0499	0.047*	
C11	0.6305 (6)	0.3689 (4)	−0.2268 (6)	0.0506 (18)	
H11A	0.5991	0.3198	−0.2544	0.061*	
H11B	0.7096	0.3766	−0.2591	0.061*	
C12	0.5423 (8)	0.4303 (5)	−0.2747 (8)	0.074 (3)	
H12A	0.4639	0.4237	−0.2425	0.111*	
H12B	0.5320	0.4265	−0.3590	0.111*	
H12C	0.5753	0.4794	−0.2528	0.111*	
C13	0.7034 (6)	0.4385 (4)	−0.0402 (7)	0.0545 (19)	
H13A	0.7097	0.4334	0.0450	0.065*	
H13B	0.6478	0.4804	−0.0608	0.065*	
C14	0.8299 (7)	0.4567 (5)	−0.0810 (8)	0.064 (2)	
H14A	0.8834	0.4137	−0.0666	0.096*	
H14B	0.8637	0.5000	−0.0384	0.096*	
H14C	0.8225	0.4679	−0.1638	0.096*	
C15	0.7989 (8)	0.2122 (6)	0.0666 (8)	0.075 (3)	
H15A	0.8652	0.1762	0.0773	0.113*	
H15B	0.7236	0.1858	0.0433	0.113*	
H15C	0.7900	0.2387	0.1393	0.113*	
H2A	0.705 (6)	0.329 (3)	−0.073 (7)	0.080*	

### Atomic displacement parameters (Å^2^)

**Table:**

	*U*^11^	*U*^22^	*U*^33^	*U*^12^	*U*^13^	*U*^23^
Zn1	0.0302 (4)	0.0493 (4)	0.0342 (4)	0.0016 (3)	0.0001 (3)	−0.0028 (3)
I1	0.0523 (3)	0.0513 (3)	0.0513 (3)	0.0029 (2)	−0.0108 (2)	−0.0073 (2)
I2	0.0678 (10)	0.0487 (8)	0.0474 (9)	0.0168 (6)	0.0150 (8)	0.0091 (5)
I2'	0.130 (4)	0.100 (4)	0.115 (5)	−0.016 (3)	−0.036 (3)	0.035 (4)
Br1	0.0832 (6)	0.0568 (5)	0.0503 (5)	0.0002 (4)	0.0141 (4)	−0.0192 (4)
O1	0.026 (2)	0.065 (3)	0.042 (3)	−0.005 (2)	0.0035 (19)	−0.009 (2)
O2	0.032 (2)	0.059 (3)	0.071 (4)	−0.006 (2)	−0.006 (2)	0.006 (3)
N1	0.024 (2)	0.035 (3)	0.035 (3)	0.0020 (19)	0.005 (2)	−0.002 (2)
N2	0.034 (3)	0.040 (3)	0.042 (3)	−0.003 (2)	0.009 (2)	0.002 (3)
C1	0.025 (3)	0.032 (3)	0.033 (3)	−0.003 (2)	0.009 (2)	0.003 (3)
C2	0.029 (3)	0.041 (4)	0.032 (3)	−0.002 (2)	0.010 (3)	0.005 (3)
C3	0.030 (3)	0.048 (4)	0.047 (4)	0.005 (3)	0.010 (3)	0.009 (3)
C4	0.051 (4)	0.040 (4)	0.041 (4)	0.008 (3)	0.020 (3)	0.008 (3)
C5	0.055 (4)	0.034 (3)	0.032 (4)	0.000 (3)	0.011 (3)	−0.003 (3)
C6	0.038 (3)	0.042 (4)	0.027 (3)	−0.003 (3)	0.006 (3)	−0.002 (3)
C7	0.024 (3)	0.041 (3)	0.036 (4)	−0.003 (2)	−0.003 (3)	0.005 (3)
C8	0.030 (3)	0.032 (3)	0.049 (4)	0.004 (2)	0.006 (3)	−0.003 (3)
C9	0.024 (3)	0.038 (3)	0.043 (4)	0.003 (2)	0.007 (3)	−0.011 (3)
C10	0.035 (3)	0.045 (4)	0.040 (4)	0.000 (3)	0.013 (3)	−0.006 (3)
C11	0.049 (4)	0.058 (5)	0.045 (4)	−0.006 (3)	0.011 (3)	0.003 (4)
C12	0.070 (5)	0.076 (6)	0.074 (6)	−0.002 (4)	−0.003 (5)	0.026 (5)
C13	0.051 (4)	0.043 (4)	0.069 (5)	−0.009 (3)	0.005 (4)	−0.007 (4)
C14	0.054 (4)	0.061 (5)	0.077 (6)	−0.019 (4)	0.008 (4)	0.011 (4)
C15	0.055 (5)	0.098 (7)	0.071 (6)	−0.005 (5)	−0.002 (4)	0.008 (5)

### Geometric parameters (Å, °)

**Table:**

Zn1—O1	1.958 (4)	C7—H7	0.9300
Zn1—N1	2.032 (5)	C8—C9	1.510 (9)
Zn1—I2'	2.545 (6)	C8—H8A	0.9700
Zn1—I1	2.5627 (9)	C8—H8B	0.9700
Zn1—I2	2.5768 (18)	C9—C10	1.536 (8)
Br1—C5	1.902 (6)	C9—H9A	0.9700
O1—C2	1.315 (7)	C9—H9B	0.9700
O2—C15	1.353 (9)	C10—H10A	0.9700
O2—H2	0.8200	C10—H10B	0.9700
N1—C7	1.278 (7)	C11—C12	1.516 (10)
N1—C8	1.478 (7)	C11—H11A	0.9700
N2—C10	1.499 (8)	C11—H11B	0.9700
N2—C13	1.503 (8)	C12—H12A	0.9600
N2—C11	1.506 (9)	C12—H12B	0.9600
N2—H2A	0.91 (6)	C12—H12C	0.9600
C1—C6	1.412 (8)	C13—C14	1.519 (9)
C1—C2	1.421 (8)	C13—H13A	0.9700
C1—C7	1.447 (8)	C13—H13B	0.9700
C2—C3	1.411 (8)	C14—H14A	0.9600
C3—C4	1.368 (9)	C14—H14B	0.9600
C3—H3	0.9300	C14—H14C	0.9600
C4—C5	1.383 (9)	C15—H15A	0.9600
C4—H4	0.9300	C15—H15B	0.9600
C5—C6	1.370 (8)	C15—H15C	0.9600
C6—H6	0.9300		
			
O1—Zn1—N1	93.11 (18)	N1—C8—H8B	109.4
O1—Zn1—I2'	111.2 (5)	C9—C8—H8B	109.4
N1—Zn1—I2'	115.1 (3)	H8A—C8—H8B	108.0
O1—Zn1—I1	115.00 (13)	C8—C9—C10	112.7 (5)
N1—Zn1—I1	109.36 (14)	C8—C9—H9A	109.1
I2'—Zn1—I1	111.9 (5)	C10—C9—H9A	109.1
O1—Zn1—I2	104.79 (15)	C8—C9—H9B	109.1
N1—Zn1—I2	111.03 (15)	C10—C9—H9B	109.1
I2'—Zn1—I2	8.4 (5)	H9A—C9—H9B	107.8
I1—Zn1—I2	120.23 (7)	N2—C10—C9	112.5 (5)
C2—O1—Zn1	120.5 (4)	N2—C10—H10A	109.1
C15—O2—H2	109.5	C9—C10—H10A	109.1
C7—N1—C8	118.9 (5)	N2—C10—H10B	109.1
C7—N1—Zn1	120.3 (4)	C9—C10—H10B	109.1
C8—N1—Zn1	120.7 (4)	H10A—C10—H10B	107.8
C10—N2—C13	110.2 (5)	N2—C11—C12	114.7 (6)
C10—N2—C11	113.6 (5)	N2—C11—H11A	108.6
C13—N2—C11	114.2 (5)	C12—C11—H11A	108.6
C10—N2—H2A	103 (5)	N2—C11—H11B	108.6
C13—N2—H2A	106 (5)	C12—C11—H11B	108.6
C11—N2—H2A	109 (5)	H11A—C11—H11B	107.6
C6—C1—C2	119.4 (5)	C11—C12—H12A	109.5
C6—C1—C7	115.8 (5)	C11—C12—H12B	109.5
C2—C1—C7	124.9 (5)	H12A—C12—H12B	109.5
O1—C2—C3	119.9 (5)	C11—C12—H12C	109.5
O1—C2—C1	123.1 (5)	H12A—C12—H12C	109.5
C3—C2—C1	116.9 (6)	H12B—C12—H12C	109.5
C4—C3—C2	122.6 (6)	N2—C13—C14	112.2 (6)
C4—C3—H3	118.7	N2—C13—H13A	109.2
C2—C3—H3	118.7	C14—C13—H13A	109.2
C3—C4—C5	119.7 (6)	N2—C13—H13B	109.2
C3—C4—H4	120.2	C14—C13—H13B	109.2
C5—C4—H4	120.2	H13A—C13—H13B	107.9
C6—C5—C4	120.4 (6)	C13—C14—H14A	109.5
C6—C5—Br1	118.8 (5)	C13—C14—H14B	109.5
C4—C5—Br1	120.8 (5)	H14A—C14—H14B	109.5
C5—C6—C1	120.9 (6)	C13—C14—H14C	109.5
C5—C6—H6	119.5	H14A—C14—H14C	109.5
C1—C6—H6	119.5	H14B—C14—H14C	109.5
N1—C7—C1	126.3 (5)	O2—C15—H15A	109.5
N1—C7—H7	116.9	O2—C15—H15B	109.5
C1—C7—H7	116.9	H15A—C15—H15B	109.5
N1—C8—C9	111.1 (5)	O2—C15—H15C	109.5
N1—C8—H8A	109.4	H15A—C15—H15C	109.5
C9—C8—H8A	109.4	H15B—C15—H15C	109.5

### Hydrogen-bond geometry (Å, °)

**Table:**

*D*—H···*A*	*D*—H	H···*A*	*D*···*A*	*D*—H···*A*
O2—H2···O1^i^	0.82	1.86	2.640 (6)	158
N2—H2A···O2	0.91 (6)	1.81 (6)	2.716 (7)	173 (8)

Symmetry codes: (i) *x*+1, *y*, *z*.
